# How Mobility and Sociality Reshape the Context: A Decade of Experience in Mobile CrowdSensing

**DOI:** 10.3390/s21196397

**Published:** 2021-09-25

**Authors:** Michele Girolami, Dimitri Belli, Stefano Chessa, Luca Foschini

**Affiliations:** 1 Institute of Information Science and Technologies (ISTI-CNR), 56124 Pisa, Italy; dimitri.belli@isti.cnr.it (D.B.); stefano.chessa@unipi.it (S.C.); 2 Department of Computer Science, University of Pisa, 56127 Pisa, Italy; 3 Department of Computer Science and Engineering (DISI), University of Bologna, Viale Risorgimento 2, 40136 Bologna, Italy; luca.foschini@unibo.it

**Keywords:** mobile CrowdSensing, IIoT, mobile edge, network mobility

## Abstract

The possibility of understanding the dynamics of human mobility and sociality creates the opportunity to re-design the way data are collected by exploiting the crowd. We survey the last decade of experimentation and research in the field of mobile CrowdSensing, a paradigm centred on users’ devices as the primary source for collecting data from urban areas. To this purpose, we report the methodologies aimed at building information about users’ mobility and sociality in the form of ties among users and communities of users. We present two methodologies to identify communities: spatial and co-location-based. We also discuss some perspectives about the future of mobile CrowdSensing and its impact on four investigation areas: contact tracing, edge-based MCS architectures, digitalization in Industry 5.0 and community detection algorithms.

## 1. Introduction

About ten years ago, the availability of the precursors of modern smartphones—in brief, devices hosting computing, communications, and sensing capabilities—boosted the birth of seminal initiatives in mobile phone sensing and, soon after that, mobile CrowdSensing (MCS) [1,2]. The core and common idea are the reliance on smartphones to sense the pulse of smart cities through the development of apps and the involvement of several users in large living labs, with studies typically lasting several months [3,4,5]. A very significant first-of-its-kind effort in this direction was the Nokia Mobile Data Challenge (MDC Nokia) that, from 2009 to 2011, involved 185 users in sensing the Lake Geneva region (Switzerland) by equipping them with a Nokia N95 phone [6,7]. The main goal of this and similar seminal works was twofold: on the one hand, to build consistent and large data sets, and on the other hand, to inspect technical issues due to the intense use of phones as sensor platforms. In particular, the MDC Nokia data set explores the possibility of collecting sensing information from personal devices, with the substantial involvement of volunteer users in a wide geographical region. The collected data come from mobile devices designed about 10 years ago, and hence is not optimized to act as a proper sensing platform. The technical issues arising from such experiment can be summarized as follows:

- The design of a mobile app able to periodically collect heterogeneous data from mobile devices. In this respect, several technical issues have to be considered, such as the non-intrusiveness of the monitoring app, the management of power consumption, the memory requirements and a broadband connection with a lower bandwidth with respect to modern network links.
- The organization of a user recruitment strategy aiming at involving the maximum number of people and at keeping them engaged during the experiment. This aspect is particularly important as it determines the success of the MCS initiative.
- The heterogeneity of the collected data: the MDC Nokia data set collected several kinds of data covering different human domains: social sensing data, location data, media creation and usage data and behavioural data.

A few years later, also motivated by the larger diffusion of more advanced smartphones available as consumer devices at a lower cost, several research projects started to address non-technical social issues also related to the active involvement of humans as enablers of this new sensing paradigm. The most distinguishing ingredient of the MCS paradigm, in addition to modern smartphone sensing platforms [8], is the crowd, that acts as a mobile data provider in urban areas. People joining an MCS data collection campaign are generally equipped with a dedicated smartphone app bridging data to and from the back-end. The app is designed to receive sensing requests to be completed; such requests are also referred to as (sensing) tasks. More advanced applications trigger tasks as soon as a device enters a specific region, e.g., a square, a street, or any generic point of interest. In this case, the back-end can selectively submit tasks only to those devices roaming in such areas or, differently, incentive mechanisms can also be applied [9,10,11].

A crucial issue of the MCS paradigm is the definition of strategies for the recruitment of volunteers and for the scheduling of the tasks assigned to the end-users as well. Of course, several strategies with different goals have been defined and tested in the last ten years of experiments, but as expected, each MCS data collection campaign operates in its own specific context that makes it differ from the other MCS campaigns in terms of the demographics, the number of users recruited, the sensing region and, ultimately, the type of data required. Significant examples of task scheduling include maximizing the quality/quantity of collected data, reducing the energy consumption of the mobile devices, or minimizing number of people involved [12].

More recently, however, researchers are considering a new methodological approach to the design of such kind of strategies that consists of exploiting knowledge of users’ mobility and sociality. In this work, we review the (re)evolution of the MCS paradigm, starting from the pioneering initiatives in mobile phone sensing conducted by Pentland’s research staff in 2004 [13], and ending with the massive data collection campaign of 2016 led by the University of Copenhagen [14] (see Section 2).

We dig into the user’s mobility by reviewing some key metrics useful for the tuning phase of an MCS initiative as well as into approaches to reveal the crowd with community detection algorithms, with the goal of capturing the dynamics of humans in their natural context. More specifically, we show social-aware and social-oblivious techniques useful for identifying groups of people linked by long-lasting social ties.

We believe that a deep understanding of mobility and sociality offers the opportunity to reshape the context regarding how to collect data with a participatory approach. Under this last aspect, we argue that spatial data about users play a key role in the possibility of making informed decisions about the scheduling of the sensing tasks.

We also note that such an understanding of users’ mobility and sociality passes through a strong abstraction of MCS data: at the bottom level, there are raw data, that are highly heterogeneous and come from different physical (e.g., GPS, Bluetooth, Wi-Fi, etc.) and logical sensors (e.g., Google Location APIs, etc.); at a higher level, it is possible to interconnect them to obtain meaningful mobility traces and then to extract useful information from them to better define both single and gregarious users’ behaviours, as well as users’ social interactions.

The mantra of the MCS paradigm is “do more with less”, meaning that it is necessary to design systems able to build high-quality data sets at low costs in terms of the number of users recruited and user’s impact. At the same time, some more recent efforts, following a similar perspective, aim to build and infer high-quality data from a large set of possibly low-quality measurements [15,16,17]. This last aspect seems to be the major barrier to the diffusion of the MCS paradigm so far. In fact, when not properly trained, end-users are sceptical about joining MCS initiatives as they are required to install a third-party application that drains their devices’ batteries and that collects “mysterious” data. Instead, the MCS paradigm requires a full and transparent involvement of the end-users to the objectives of the experiment, that is, collecting data to understand the complex dynamics of people in urban environments. The recent contact tracing app designed to limit the COVID-19 pandemic demonstrates an ineluctable example of the benefits of the participation of a massive crowd. The more people who allow to app to track their contacts, the higher the possibility of interrupting the never-ending contagious chain. Under this aspect, we take into account the fact that contact-tracing apps are a very specific use case of the MCS paradigm, in which the data set to be collected is the temporal evolution of social contacts.

We briefly review some notable MCS experiments conducted in the last 10 years by focusing on some qualitative markers showing the growing scale of the experiments, as reported in Section 2. We then focus on how mobility can be analysed and how communities can be detected with two representative approaches, spatial and co-location-based as reported in Section 3. Lastly, we frame four application scenarios that highlight the exploitation of the techniques we reviewed with an outlook to the near future in Section 4.

## 2. A 10-Year Journey in Mobility Analysis

The optimization of an MCS data collection campaign requires the initial understanding of how people move and how they interact. Such a level of understanding opens up the possibility of optimizing several aspects, such the selective assignment of sensing tasks to the end-devices, the assignment’s scheduling during the day and the identification of geographical areas covered by the data provided by the users’ devices.

Recent works addressed the goal of understanding and modelling human mobility in urban areas [18,19,20,21,22] at different depths. Such kinds of break-through analyses are possible thanks to the collection of massive mobility data sets that realistically reproduce mobility flows at high-resolution temporal scales. The accuracy of these data sets, however, greatly depends on the technology adopted to localize the users.

Human mobility is traditionally studied by analysing Call Detail Record (CDR) and GPS logs [23,24,25,26]. CDRs report information about the incoming/outgoing calls of a device. The information generally exploited for the mobility analysis includes the timestamp, the cell tower to which the device is connected with, and the duration of the call. As a result, the location of a device is inferred by using the location of the connected cell tower. A CDR-based data set approximates a significant sample of a population well, since mobile devices are extremely diffused. Furthermore, no action is required from end-users, as the CDRs are generated by the network providers [27]. However, such approximation leads to a possible inaccuracy of the data set. Indeed, a device might be linked with a cell tower far away from the device itself. In addition, the device position can be estimated only during an incoming or outcoming call, while the position is not known during time periods of inactivity of the device. We also observe that such data sets are generally not publicly available, since their analysis can offer strategic information typically preserved by the network providers.

Human movements can also be studied by analysing GPS traces provided by wearable devices, such as smartphones, tablets, and smart watches [28]. GPS traces report the device position with a reference system (e.g., WGS84 coordinates) and with higher accuracy than inferences obtained with CDR logs. The accuracy of the GPS technology in outdoor environments is generally bound in the order of a few meters, while in indoor environments, the accuracy rapidly decreases, since the strength of the GPS signals tends to reduce or completely disappear. Differently from CDR logs, the time resolution of GPS traces depends on the update frequency of the device: the higher the frequency, the higher the accuracy of the collected traces. However, the GPS traces require the active involvement of the devices to perform the localization and to upload the position to a back-end server.

We finally mention a third option to construct a mobility data set, namely the use of information obtained from Social Networks (SNs). These traces are also referred to as Location-Based Social Networks [29,30]. The majority of SNs enable the possibility of localizing their users when they check-in or when they check-out of a point of interest. The location of a device can be determined not only with GPS, but also with the location information provided by the Wi-Fi hot spots and probes [31] or cellular base stations, or with a combination of them all. The major drawback of SN-based solutions derives from the fact that only specific kinds of places are monitored (e.g., restaurants, shops, museums, etc.); therefore, it is not possible to constantly track users as is possible with GPS traces.

For these reasons, we focus on GPS-based data sets, and we describe a 10-year-long journey of initiatives devoted to collecting mobility data sets, with the goal of understanding, with a data-driven approach, the all-human tendency to move and interact with others. We restrict the scene to six experiments ranging from 2004 to 2016 based on GPS traces gathered with mobile devices of different historical periods. For each of the data sets, we consider three metrics which provide a first rough indication of the quality and representatives of the data set. The described metrics are extracted from the meta-information released with the data set or by analysing the data set itself. The considered metrics are:

- The number of recruited or monitored users: the number of users varies along with the time, but we consider the maximum or the declared number of users participating in the MCS initiative.
- The duration of the experiment expressed in months: the duration refers to the time elapsed from the first to the last observation.
- The types of data collected: this information describes the number of different data types provided by the data set; hence, it provides a measure of the variety of the collected information. As a representative example, we consider GeoLife, which provides three types of information: timestamp, device coordinates (latitude and longitude) and the altitude (we do not consider the Transportation mode labels in this case, which are not always available, as described in [32,33]).

**Figure 1:**
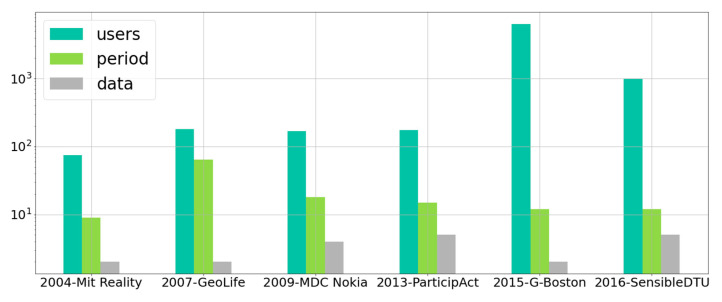
Evolution of the mobility data sets in terms of: number of users, time duration and heterogeneity of collected data.

We compare the six experiments with respect to the described metrics in order to show how they evolve along with the time. Figure 1 reports such evolution from which we observe two main trends: The increasing number of users recruited and the improvement of the sensing capabilities of the devices. Their combination led to an increase in the heterogeneity of the gathered data, as described in the following.

The Reality Mining Data Set [13] involved about 75 students or faculty in the MIT Media Lab, Boston, MA, USA. Users were equipped with a Nokia 6600 device able to detect proximity with nearby devices by using the Bluetooth discovery protocol. The data collected comprised call logs, Bluetooth contacts, localization, identifier of the cell tower, usage the apps and phone status. The GeoLife data set [32,33] was organized by Microsoft Research Asia, with about 182 users in very long period, ranging from April 2007 to August 2012. The data collected only included the device position (latitude, longitude, and altitude) and, in some cases, the type of transportation used by the volunteer users. The devices used varied from GPS tracker to GPS phones, with most of them being located in the Beijing area. The MDC Nokia [6,7] was the result of a joint initiative between Idiap and NRC-Lausanne from 2009 to 2011. The data set involved about 200 users of different demographics moving in the Lake Geneva region, Switzerland. The data collected comprised the device’s locations, SMS, calls, picture photos, devices in proximity detected with Bluetooth discovery protocol, Wi-Fi scans and several sensing data such as accelerometer, audio samples, calendar, etc. ParticipAct [34] was a pure MCS living lab designed by the University of Bologna, Italy. The project involved about 180 students roaming in the Emilia Romagna region, and mostly moving in the Bologna area. The data collected included device position (i.e., latitude and longitude), and data about the task’s competition such as pictures, feedback, questionnaires, etc. Students were equipped with a smartphone and the location was obtained by combining different sources of information. The authors of [17] describe a massive passive data collection study in the area of Greater Boston, MA, USA (G-Boston). G-Boston aimed to quantify the degree of asymmetry of the followed users’ routes, to compare and estimate those factors influencing the choice to change route and finally “assessing the veracity of passively collected smartphone data to understand pedestrian route choices”, as reported in [17]. Data were collected with a smartphone-based mobile application designed to track the daily routines of the participants. The app passively collected the users’ movements by tracking the device position. Positions were obtained by exploiting assisted GPS, the tower-based position of the network operator and satellite-based GPS. The collected data covered a period of 12 months (15 May 2014 to 1 May 2015), with a total of 263.670 trips (filtered down to 103.835) from 6.424 unique users. Finally, the SensibleDTU large-scale experiment [35] was organized by the Technical University of Denmark and the consortium of researchers from the University of Copenhagen. The project organized two data collection campaigns, scaling up to 1.000 volunteers (mostly students). The data collected included device positions and devices in proximity detected with Bluetooth, as well as different kinds of Wi-Fi data.

In the next sections, we focus on two orthogonal aspects that can be leveraged to optimize the design of an MCS data collection campaign, namely measuring features of human mobility (Section 2.1) and extracting information about people’s contacts from the mobility traces (Section 2.2).

### 2.1. Extracting Mobility Features

For the purpose of this section, we consider an analysis of a subset of the mobility metrics of interest for the MCS objectives. The metrics analysed are obtained from the ParticipAct data set [34], a representative MCS experiment conducted by the University of Bologna in 2014.

**Figure 2:**
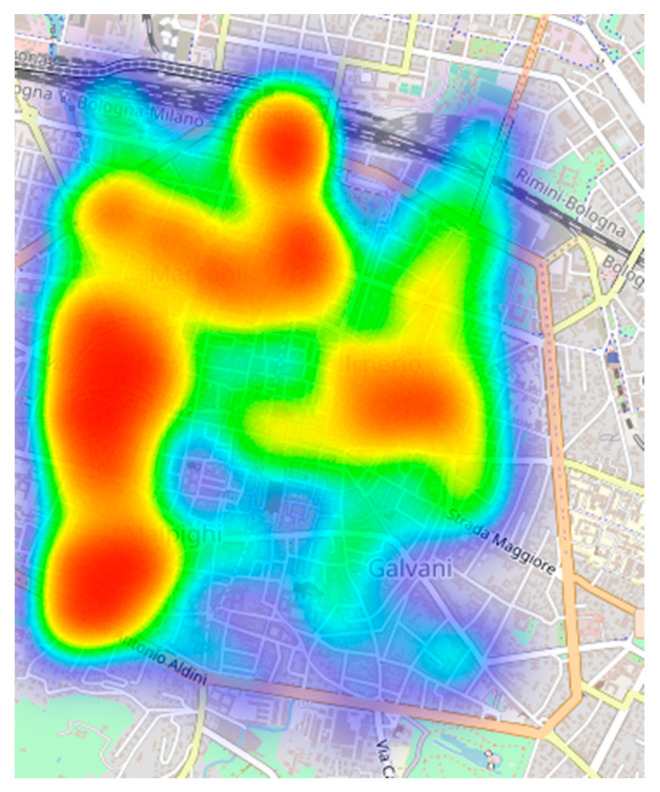
A graphical representation of ParticipAct data set.

**Figure 3:**
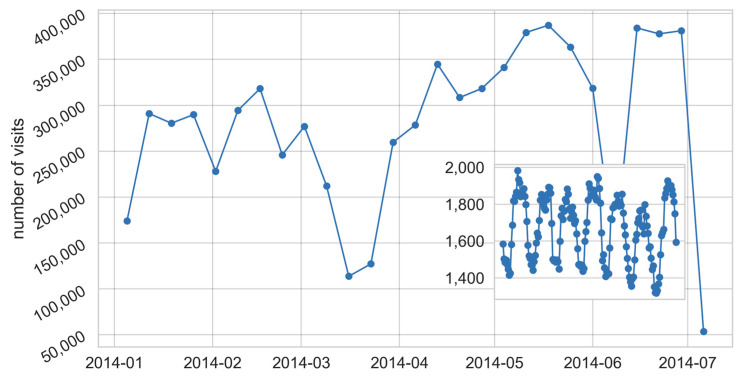
Number of visits during 7 months of mobility for ParticipAct data set. The graph reports the time periods with high and low numbers of visits. The inset graph shows a typical pattern of visits for a representative week.

A typical MCS architecture requires remote servers to periodically assign tasks to users. Tasks can directly involve the users (e.g., providing feedback or answering a questionnaire), or they can be autonomously completed without any human intervention. In this respect, it is worth noting that tasks that do not require any intervention from the user allow one to collect sensing information from a device, e.g., collecting GPS position, sampling ambient light, measuring noise intensity or assessing the signal strength of Wi-Fi networks. In both cases, it is important to schedule the right task at the right moment so as to increase the probability of successfully retrieving data from the target users. Furthermore, we also consider the possibility of sending so-called geo-referenced tasks to users. These are tasks that can be completed by users roaming in a specific geographical region and that can be completed in a certain time period. As a representative example, we refer to the possibility of collecting pictures from a historical square for one week. To this end, understanding the pattern of visits of volunteer users might ease the task of scheduling. It is worth noting that the metrics described in the remainder of this section are computed by analysing all the data available for a considered snapshot of length Δ*t*. As a result, the possibility of predicting the user’s behaviour in terms of the analysed metrics is based on the assumption that users will act similarly in the next snapshot. In Figure 2, we report a graphical representation of ParticipAct and in Figure 3, the aggregated time series of the users’ visits monthly.

The graph shows a significant variation in the number of visits during the year. We observe time periods during which users move more frequently (e.g., April to May 2014) and other periods when the mobility slightly decreases (e.g., late in 2014). The inset graph in Figure 3 further details the users’ visits on an hourly basis. From the inset graph, it is possible to observe the daily pattern of mobility, e.g., working hours followed by off-time hours. Such information can be exploited in order to predict the optimal time period for the assignment of tasks to the end-devices. As a representative example, we refer to a MCS data collection campaign designed to submit tasks to users only during working days in the hour range of 9 AM–6 PM.

**Figure 4:**
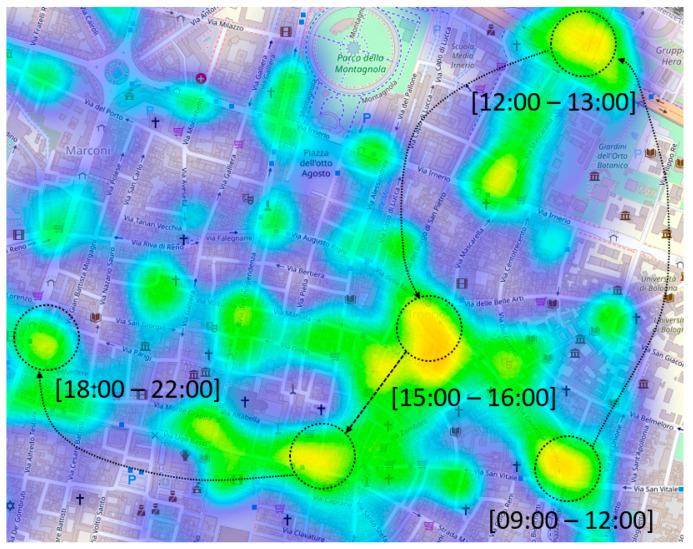
Graphical representation of stop-places for ParticipAct data set. The heatmap shows the stop-places and the time of day such places become active.

Task assignment is also influenced by the locations that the users visit most. More specifically, we argue that spotting crowded areas helps to give tasks to those users visiting high-density places at the right time, such as, e.g., train stations, squares, or other points of interests. In Figure 4, we report a heatmap showing two detailed levels: on the one hand, it shows the top-most crowded locations, and on the other hand, it shows when such locations become active in terms of users’ visits. High-density locations are identified with the method proposed by Hariharan and Toyama (2004) and implemented with the Scikit-mobility library [36]. In particular, given a geo-referenced data set, we identify the so-called stop-location characterizing regions where users spend x minutes within a distance of y km from a given trajectory point. Such regions are identified by considering the median latitude and longitude of the user’s previously identified stop-locations. The black arrows reported in Figure 4 show when such regions become active along time. More precisely, it is possible to identify the stop-locations on an hourly basis for one working day, so as to obtain 24 distinct snapshots and recombine them by only linking together the top-most crowded locations.

Finally, we mention the possibility of understanding, in an aggregated and anonymized way, the mobility features of the users. In particular, we refer to the geographical distance travelled by users. Certain users tend to commute regularly, following repetitive flows (e.g., home to work, home to school, etc.), while other users explore new locations far from their homes. The authors of [19] investigate the impact of “recurrent mobility” with a GPS-based data set. Being able to profile the users in terms of such mobility attitudes allows one to select the best target users for a certain task. For example, if the goal of an MCS task is to monitor some environmental parameters in a bounded region during working hours, then the users to consider are those visiting such regions on a regular basis. In this respect, two metrics are useful to characterize such features:

- The radius of gyration, *r_g._*
- The maximum travelled distances of users, *M_d_*.

It is worth noting that the time period used to compute the two aforementioned metrics greatly affects the obtained results. Our objective is to characterize users during a significant time period, e.g., months, so as to capture their mobility patterns. To this purpose, the two metrics are computed by analysing all the mobility traces for the considered data set so as to extract a pattern useful for planning an MCS data collection campaign.

**Figure 5:**
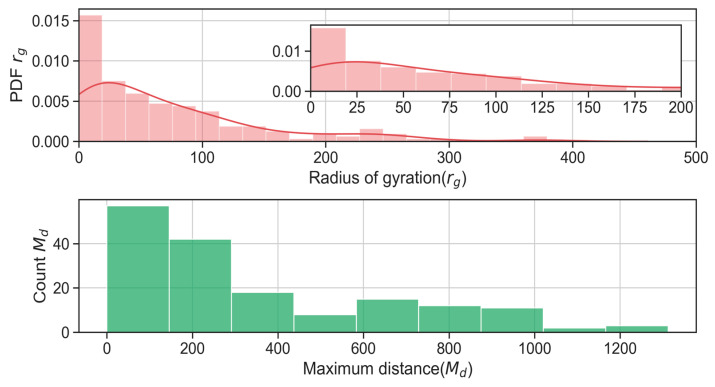
Radius of gyration and distance travelled by users for ParticipAct data set.

More specifically, the radius of gyration quantifies the “typical distance travelled by an individual” [19], providing an indication of the mobility pattern of a given user. The radius is computed as follows, as implemented in [36]:

$$r_{g}=\sqrt{1/N\sum_{i\in L}n_{i}{\left(r_{i}-r_{m}\right)}^{2}}$$

where *L* is the set of locations visited by a user, *r_i_* are the coordinates of the *i*-th location, *r_m_* is the centre of mass of the user’s trajectories, *n_i_* is the visitation frequency of the *i*-th location and *N* is the total number of the user’s visits. In Figure 5, we report the two aforementioned metrics.

The distribution of *r_g_* peaks around 25 km, which indicates that, on average, users tend to travel in a radius of 25 km from their centre of mass, e.g., their home location. Moreover, the ParticipAct users mainly take trips at a maximum of 0–100 km from their home location, as reported in the histogram of *M_d_*.

**Figure 6:**
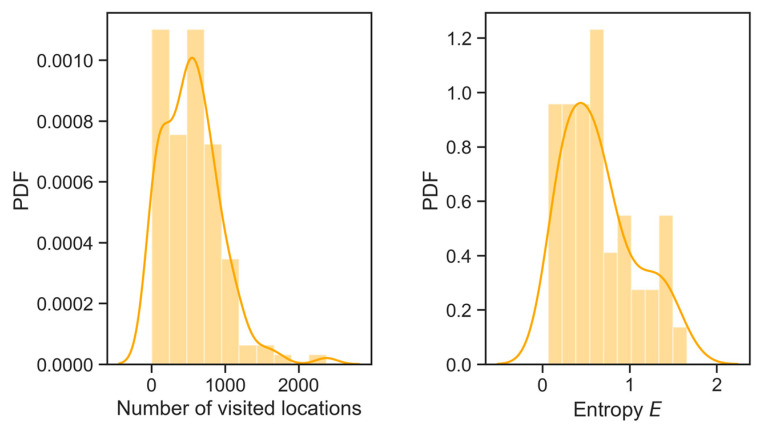
Locations visited and real entropy for ParticipAct data set.

**Figure 7:**
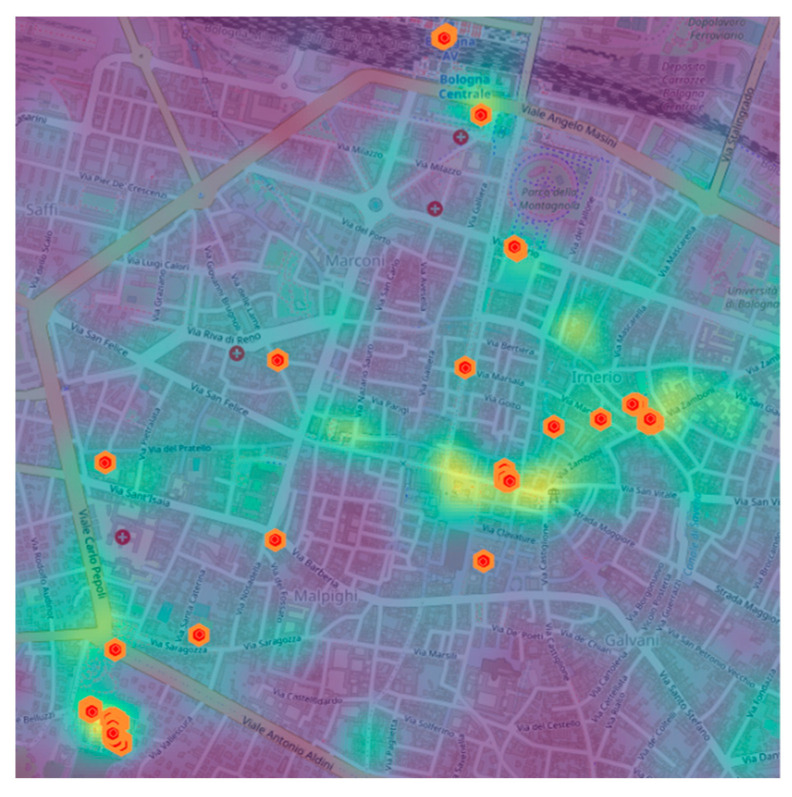
Graphical representation of location entropy for ParticipAct data set.

Concerning the possibility of predicting the users’ locations, it is possible to compute the distribution of the locations visited and the real entropy, as reported in Figure 6. In the first graph, we report the distribution of the number of locations users visit, while in the second graph, we report the real entropy, E. The purpose of the real entropy metric is to measure not only the frequency of visitations, but also the order in which locations are visited so as to capture the “the full spatiotemporal order present in a person’s mobility patterns” [37]. More specifically, for the ParticipAct data set and for the selected period, we compute the real entropy, *E*, for each of the users and in Figure 6, we plot the probability density function (PDF) of the observed values. From the distribution, we observe that the mean value of E ≈ 0.6, meaning that the next location of a randomly chosen user can be found in 2^0.6^ ≈ 1.51 locations. In other words, and only for the data set considered, it is possible to predict the next location with relatively high accuracy. This kind of information can in turn be exploited to figure out the visited locations and hence to plan a data collection campaign from a set of locations. We further analyse the potentialities of predictability metrics, and we also analyse the location entropy of the visited locations. More specifically, from the considered data set, we extract all the locations visited at least once by the end-users, namely the set *L_E_*. For every location, *j* ∈ *L*, we then compute the random location entropy, *L_E_*(*j*)*,* which measures the predictability of *j* under the assumption that each user visits *j* with the same probability. The location entropy is computed as follows (as reported in [36]):

$$L_{E}\left(j\right)=log\left(N_{i}\right)$$

where *N_j_* represents the number of distinct users visiting *j*. In Figure 7, we report a heatmap showing the *L_E_*(*j*) value for all the considered locations. From the figure, it is possible to clearly identify highly predictable locations (marked with yellow gradient); hence, such locations can be considered as targets for an MCS data collection campaign.

It is worth pointing out that the effectiveness of the above user location prediction process depends on the accuracy of the mobility information in use. The ParticipAct data set provides mobility information with a time resolution of 2.5 to 5 min. Such a sampling rate can vary according to several factors, such as the absence of location information, unavailability of the device and the presence of energy-saving applications preventing the use of Wi-Fi or broadband connections. Furthermore, the accuracy of the user’s location depends on the quality of the GPS signal, or the location information used to localize the device. It follows that the higher the sampling rate and the accuracy of the user’s locations, the higher the quality of the extracted mobility features.

### 2.2. Extracting Co-Location Traces from Mobility Traces

Mobility is not the only feature of interest for the design of an MCS data collection campaign. An orthogonal dimension of humans is extracting information about the temporal dynamics of people. In this section, we cover features of interest for the MCS scenarios that are derived from the study of co-location traces. Users are co-located when they are in the same place at the same time. Co-location can be studied by analysing co-location traces which, generally, report the timestamp and the users in contact. Such traces can be obtained either with proximity sensors or by processing mobility traces. In the first case, the trace is obtained as a direct output of the sensor used. Examples are the Bluetooth Low Energy (BLE) protocol and the Radio-Frequency IDentification (RFID) technology that allow one to periodically scan the surrounding environment and to track proximity with nearby devices in the range of few meters (from distances of 0 m to 10 m). We refer to the SocioPattern (http://www.sociopatterns.org, accessed on 1 September 2021) project for a high-quality collection of co-location data sets.

Data are collected with RFID sensors capable of detecting proximity between users in the range of 1 m to 1.5 m (approximately) at a high temporal rate. In the second case, co-location is obtained indirectly by analysing the position of the user’s devices and by detecting those in the range of Δl meters for at least Δt seconds. Obviously, such inference is prone to errors as location inaccuracy can lead to false positive and false negative proximity between users’ devices.

Given the co-location trace, the analysis of some temporal features of the device contacts reveals interesting properties. The literature in this area typically focuses on three state-of-the-art metrics [38]: the distribution of the inter-contact time, the distribution of the contact duration, and the number of contacts. Given a pair of individuals, the inter-contact time measures the time elapsed between two successive contacts of a dyad. The shorter the inter-contact time, the more frequently a dyad meet. Contact duration measures how long contacts last. Long-lasting contacts generally give rise to voluntary interactions, while short contacts are typical of involuntary gatherings. The number of contacts measures how many contacts an individual has with others. The number of contacts characterizes the social attitude of a person. More specifically, users with a high number of long-lasting contacts are typically gregarious individuals. It is worth pointing out that in MCS, terms such as long-lasting contacts and gregarious behaviours are referred to as contacts and bonds between users that do not necessarily share a parental relationship but habitually stay for a relatively long period of time in proximity with each other. It follows that such meanings are intended in a broad way, also considering the relationships between strangers that share common habits as gregarious. Examples include students that wait at the bus stop for the school bus daily, or workers committed in a common long-term project. The examination of the routine of contacts allows for the detection of co-location information between individuals.

Co-location traces also allows one to reveal *communities*, namely a group of users proxied by their end-devices linked with a high number of contacts. Such structures can be detected with community detection algorithms designed to capture the gregarious attitudes of humans.

## 3. Finding the Crowd with Community Detection Algorithms

People generally exhibit gregarious behaviours. In fact, we tend to establish relationships with others according to the natural attitude of preferring “similar” ties instead of dissimilar ones [39,40]. Similarity is also referred to as *homophily*, namely the tendency of people to bind with others with similar profiles such as, e.g., interests, visited locations, acquaintances, or opinions. 

Homophily shapes our social relationships in many contexts, such as our working environment and private life. Being able to spot similarities between individuals and cluster them is not an easy task. To this purpose, in this section, we report some results of the vast literature investigating community detection strategies that can be adopted to optimize an MCS experiment [41]. The interested reader can find two general and comprehensive surveys of community detection algorithms for general networks in [42] and [43]. Indeed, an MCS data collection campaign might require the collection of data from specific target users, such as students of a specific academic year, commuters sharing public transport, or colleagues working in the same office.

The current literature reports several definitions of the term community, as the concept itself may assume different connotations based on the domain it is applied in [41,44]. As a result, many community detection algorithms are available, with the goal of detecting clusters of nodes by analysing different features of the network. In the next subsections, we report a subset of the methods available, algorithms based on the analysis of the locations visited by the users (see Section 3.1) and algorithms based on the analysis of the contacts between them (see Section 3.2). In the first case, the goal is to identify clusters of users roaming in a specific location for a time period (also referred to as stop-place detection), without considering the ties binding them.

**Figure 8:**
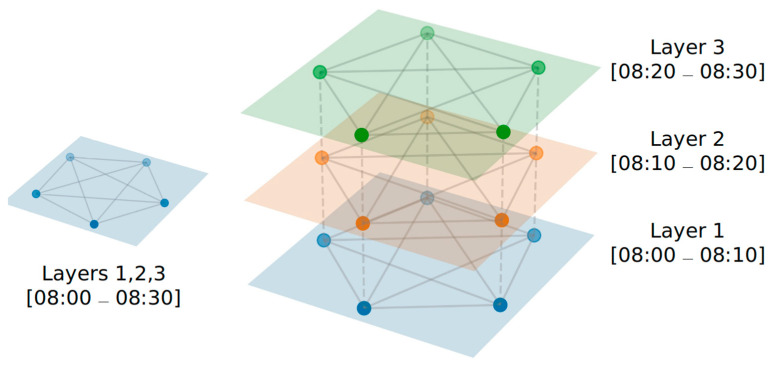
Representation of a single and of a multi-layer network. With a single network layer, the temporal dimension is not considered, giving rise to a single connected component spanning for 30 min. Instead, the multi-layer network shows the active links during each of the 3 layers of 10 min duration.

Differently, in the second case, the goal is to detect clusters of users by analysing the number, magnitude, and links among them. More specifically, we focus on those algorithms able to analyse dynamic networks, namely networks with links connecting nodes that appear and disappear as time progresses. Figure 8 shows an example of such dynamic evolution. This feature is extremely important for the MCS domain, since communities may evolve over time, creating, splitting, merging, destroying, and creating again.

### 3.1. Spatial-Based Approaches

Among the main community detection algorithms that only exploit spatial information, DBSCAN [45] represents a popular approach. DBSCAN (Density-Based Spatial Clustering Algorithm with Noise) is an unsupervised learning approach used to identify clusters; it is possible to also adopt such an approach to detect communities of users’ devices by identifying high-density locations. DBSCAN identifies groups of points (i.e., clusters) in proximity in the space. Typically, for community detection purposes, the points represent the locations visited by the users and they are identified by geo-spatial coordinates. DBSCAN detects clusters of points using two main parameters: the spatial density and the distance threshold. The spatial density sets the minimum number of points required to form a cluster, while the distance threshold determines the maximum distance for the algorithm to identify two points as belonging to the same cluster. DBSCAN computes the distance between all the points and assigns a label to each cluster. Another spatial-based approach for community detection is given by partitional algorithms, among which one of the most known is *k*-means [46]. Briefly, *k*-means is a spatial clustering algorithm and one of the simplest unsupervised learning approaches based on partitions. It operates by first splitting *n* observations into *k* clusters and by assigning the observations to each cluster according to a given heuristic. Afterwards, it computes the centroid of each cluster and creates new partitions by reassigning each observation based on the nearest average distance of the observation from the cluster centroid. The procedure is iterated until the algorithm converges. The objective of the clustering performed with *k*-means is the minimization of the total intra-cluster variance.

We also mention an interesting algorithm to identify the stop-places, namely locations where users stop for a while. InfoStop [47] implements a stop-place detection algorithm that overcomes the limitations of the DBSCAN. InfoStop implements a flow-based approach by leveraging the network community detection algorithm InfoMap [19,20]. The identification of the stop-places consists of two steps: it firstly labels each of the user’s trajectories as a trip or stay trajectory. The second step aims at clustering the points previously detected by using the InfoMap algorithm (see Section 3.2). In particular, the network generated is composed by nodes representing the trajectories labelled as stay (first step) and links connecting the nodes are added if a pair of nodes are at a given distance.

### 3.2. Co-Location Based Approaches

A different approach is adopted by community detection algorithms that leverage the co-location information between users. As previously reported, we focus on some algorithms able to detect communities on dynamic networks. Figure 8 reports an example of a dynamic network represented as a multilayer network, where each layer of the network reports the contacts between nodes (e.g., the user’s devices) in a time bin of Δt (in the figure, each time bin corresponds to 10 min). Nodes can be linked inside each layer (intra-layer links) and across different layers (inter-layer links). For example, node *x* is linked with node *y* from layer 1 to layer 2, meaning that a link spanning two different layers exists. In this section, we describe three algorithms designed to detect communities by considering such dynamic structures.

Two interesting social-based approaches to community detection are represented by TILES (Temporal Interactions Local Energy Strategy) and iLCD (intrinsic Longitudinal Community Detection) [48,49]. The former implements an online iterative procedure and explores the flow of interaction between people through a domino-effect strategy and a label propagation procedure. The latter recreates the network of contact by processing each chronologically ordered piece of co-location information step-by-step and then determining new communities based on a minimal community pattern (e.g., a 3,4-clique).

The InfoMap [14,50] algorithm can also be adopted to detect communities with time-evolving networks by exploiting the multilayer network support (see Figure 8). InfoMap detects such flows connecting nodes within and across different layers. In particular, the algorithm runs a random walker, visiting nodes with a probability given by links connecting nodes. The walker periodically performs a jump to other nodes and a jump to other layers. As a result, the random walker visits the whole network spanning across multiple layers. However, the walker more frequently visits tightly connected nodes and thus remains “trapped” in clusters of nodes with a high number of connections. The random walker describes the path followed by using codewords based on a set of codebooks. The goal of InfoMap is to compress such description by minimizing the map equation [50] framework. As a result, the problem of detecting communities is solved my minimizing the length of a set of codewords describing the visits performed by a random walker.

Communities (or clusters) can be measured to characterize very different properties, such as cohesion, the amount of information represented, the stability of the partitioning, etc. [51]. A first aspect to be considered is the existence of the ground truth, namely the real structure of the communities. If this information is available, then it is possible to compare how much the algorithm used to detect the communities reflects the real structure. Under this aspect, we refer to the Adjusted Rand Index, a family of metrics based on Mutual Information, and metrics assessing the homogeneity and completeness of the communities.

These representative examples allow one to compare the difference between what is obtained from what is expected. Unfortunately, the ground truth is not always available. In these cases, some metrics can still be used to assess the properties of interest in the detected communities, for example, how much communities overlap, how stable they are along time or how free they are from noisy data, etc. As a result, we argue that it is not possible to provide a rule of thumb for a quantitative assessment of communities; rather, its evaluation highly depends on the scenario considered.

## 4. Usage Scenarios and Beyond

The information regarding the interaction among volunteers of the MCS platform and their communities can be exploited in several ways. Here, we review four scenarios for the use of this information, namely contact tracing with community detection, edge selection, and finally, prediction of the behaviour of communities, starting with the most realistic and perhaps current, up to the most futuristic.

The first scenario (contact tracing with community detection) emerged with the recent COVID-19 pandemic as a means to prevent the diffusion of the epidemic through the active contribution of the crowds; the second aims at optimizing the MCS platform itself and emerged in the recent scientific literature; the third scenario refers to the possibility of improving digitalization applied to manufacturing processes. Finally, the last scenario may also be applied to the optimization of the MCS platform itself, but it may adapt to new application scenarios.

### 4.1. Contact Tracing and Community Detection

The first usage scenario concerns the use of contacts among users and of communities to the purpose of finding spatial relationships among users. The recent COVID-19 pandemic has seen a rise in the use of large-scale apps for contact tracing, as a means to automatically track infected people [52,53]. Such apps are an interesting example of the MCS paradigm, as users’ devices periodically upload their contacts with proximity-sensing interfaces. The analysis of collected data is extremely useful to re-build the contact network, with higher accuracy than self-reporting tools (e.g., questionnaires or surveys) [52,54]. In this respect, we consider the possibility of employing predicting tools to infer information useful for tracing contacts and to detect communities as a valuable option [55]. More specifically, we refer to the possibility of:

- Identifying communities of users that regularly meet;
- Predicting the next visited community and the location of such meeting.

We argue that these kinds of techniques increase the possibility of improving prevention policies limiting or controlling contacts in crowded areas. The goal is not to track a single user, but rather to observe the benefits of the actions on a large population on a higher scale. Similarly to some studies showing the impact of a lockdown period to the vehicular and pedestrian mobility [56], it might be possible to study how users’ mobility and sociality changed before/after a quarantine period, by analysing, e.g., the reduction in contacts and the contraction of communities and locations where such contacts occur. Concerning the large variance introduced by such tools, we acknowledge that this might represent an issue. However, all statistical tools inherit a certain degree of inaccuracy. Our opinion is that the quality of the data set, the demographic, the duration and the performed analysis are all factors that can contribute to limiting the inaccuracy of the predictability. A further step to support the prevention of COVID-19 disease consists of determining communities of users to analyse the spread of infection spread within a community over time, as suggested in [57]. Moreover, understanding the membership of humans to communities, the spatial scale of their human interactions, and how the network of human communities’ changes over time is a powerful tool to target interventions to at-risk areas without unnecessarily restricting areas at low risk of resurgence [58]. Even if a user does not interact with all the community’s members, the domino effect might quickly spread a virus to all the members in short time. Such diffusion might be brutally aborted with an aggressive policy that alerts all the members of an infected subject in advance. A key aspect in this scenario is the number of users participating to the MCS campaign, which, if too low, may prevent the effectiveness of contact tracing. Beyond common strategies for users’ recruitment [59], a different approach in this regard comes from social amplification strategies [60] that aim to implicitly collect data from non-subscribers. In this scenario, each subscriber implements the concept of social amplification by collecting, by means of its sensor or interfaces (such as smart watches and smart devices), information about the presence of other people in its surrounding, thus enabling the construction of aggregated data about gatherings (e.g., the construction of temporal maps of the crowds).

### 4.2. Extending the MCS Architecture with an Edge Layer

A traditional MCS architecture implicitly assumes that data provided by user’s devices can be gathered with an end-to-end connection (e.g., 5G broadband links) between the device and the back-end server located on the cloud.

Most recent deployments, however, also introduce an additional, intermediate edge layer between the back-end and the end-devices. Such a layer has the goal of easing the data collection and the distribution of tasks to the end-devices with a bridge to/from the back-end. This edge layer is deployed in proximity to the base stations providing connectivity, so as to be close to the end-devices and hence to reduce the communication latencies. Such a paradigm is also known as Multi-access Edge Computing (MEC) [61].

A further enhancement of this architecture, which has been envisioned in several recent works [38], consists of configuring some of the end-devices to act as (mobile) edges themselves. Indeed, Multi-access Mobile Edges (M2ECs) of a MEC architecture are regular end-devices that exploit opportunistic communications with other devices to perform two basic operations: retrieving data to be uploaded to the back-end and propagating MCS tasks to the end-devices. The two operations can be achieved by exploiting wireless short-range ad hoc communications through, e.g., direct Wi-Fi, Bluetooth or ultra-wide bands, short-range links that generally are free-of-charge. This enhancement trades the costs of broadband communications (both in terms of energy and subscription costs) with an increased latency in the communications between the end-devices and the back-end servers, and it is suitable for applications that require an off-line analysis of collected data.

One of the key issues of such layered architecture is how to select the end M2EC devices among all the devices of the MCS subscribers [62]. Clearly, the selection of M2ECs should be carried out in such a way to increase the opportunities of (short- range) communication between them and the other end-devices. In this regard, a consolidated approach consists of exploiting the knowledge of MCS user communities to identify the leaders that may act as proxies for the members of the community they represent [62]. Since members of a community interact periodically, the resulting dynamic network can be analysed to measure the centrality of its members (e.g., betweenness, eigenvector, k-core scores). The higher the score adopted, the more “central” a device is to the communications among members of its community [44].

### 4.3. Digitalization and Industry 5.0

The possibility of retrieving data from IoT-ready devices is enabling the digitalization process envisioned with Industry 5.0 [63], and consequently, the MCS paradigm can also fit industrial scenarios. An example is the increase in (indoor and outdoor) safety policies. In fact, the sensing information retrieved from wearable units might also refer to the proximity between employees and specific machinery. Such information allows one to recognize hazard situations and to adopt policies of prevention [64]. Similar considerations can also be applied to situations in which it is required to monitor the occupancy of specific locations, such as data centres or production sites [65]. In these cases, the MCS paradigm allows one to increase the amount of useful information so as to determine whether such environments are occupied and how people move inside such places. In this respect, we mention the crowd localization [31,66] as a concrete application of the MCS paradigm. The idea is to improve the accuracy of an indoor localization system by exploiting data retrieved from target users. This approach can also be applied to industrial settings, in which employees are equipped with sensing tags able to localize and to detect nearby objects.

The MCS paradigm provides a valuable approach to environmental monitoring as well. Indeed, the sensing capability of wearable units, such as smartphones and wristbands, opens up the possibility of implementing continuous, participatory monitoring [25]. Of course, the accuracy of the collected data might be affected by disturbing factors. However, the aggregation of such data still represents an added value.

### 4.4. Predicting Community Behaviour

MCS platforms traditionally combine information about the mobility of their subscribers along with their profiles, to plan and activate data collection tasks [12]. This is particularly useful in campaigns that collect geo-referenced data. For example, a task or a query can be directed to subscribers that are active in a specific area or that are likely to reach a given location. However, as discussed in Section 3, more sophisticated information about the dynamics of MCS subscribers (such as the identification of communities of subscribers) can emerge from the analysis of location information of the subscribers and/or from information about their reciprocal proximity. In perspective, this information may be enriched with predictions about the behaviour of communities such as, e.g., future stop-places, the turnover of members, lifetime, etc. In this respect, it is worth noting the use of predictive methods also based on neural networks, e.g., LSTM (long–short-term memory) or GRU (Gated Recurrent Units) able to identify recurrent patterns and to predict, with reasonable accuracy, the next visited location, or the next stop location [37,67]. This kind of information creates new perspectives in the planning and organization of MCS data collection campaigns that currently exploit information regarding existing communities, but that, in perspective, may benefit for scheduling purposes, coverage and in general, from improving the efficiency of the campaigns of predictions about how communities behave and evolve. Another potential application of the MCS paradigm may come from the analysis of the mobility of community members, with the goal of identifying crowds. This task is currently achieved by using cameras and image processing [68], but a similar analysis could be conducted by injecting suitable tasks in an MCS platform, so as to obtain the crowd density coverage of large public events, for instance, or for security applications. Although this approach has still not been explored to our knowledge, some recent approaches of community density identification such as [69] may suggest this as a viable direction. Finally, coverage is another key-objective for the MCS domain since, often, it is required to collect data from a bounded geographical area. Such a goal can be obtained by better understanding how people move and, more specifically, how clusters of people visit the environment [44].

## 5. Discussion and Conclusions

The effectiveness of an MCS data acquisition campaign strongly relies on the active involvement of the end-users. The higher their active contribution is, the higher the quantity of data that can be collected is. In this respect, an important contribution to the data collection process, which, however, is not explicitly produced by the users, comes from their mobility and from their implicit network of relationships in the form of contacts, communities, and their dynamics.

We have extensively discussed how such data can be processed by community detection algorithms designed to detect clusters of people linked with robust ties, and how they can be used in three application scenarios. From these perspectives, online co-location-based approaches seem to return more accurate information on the inner circles of the users if compared with the ones obtained by community detection algorithms based on the comparisons of spatial information belonging to single individuals. Notwithstanding, both online co-location-based and spatial-based can result in valuable approaches for off-line mobility data analysis. Concerning these scenarios, we note that they are representative of two needs: one of the MCS platform itself that uses mobility data, contacts, and communities to optimize its internal mechanisms; and one of the applications that use the same kind of information for their own purposes. However, there can be further scenarios that could be considered with respect to the MCS data collection campaigns, and a plethora of community detection approaches or artificial intelligent techniques exist, devised to discover more or less strictly connected clusters of people by exploiting the information hidden within the users’ mobility data. Concerning the need to optimize the MCS platform itself, we believe that the increasing diffusion of the MCS paradigm will give rise to massive data collection campaigns. Incentives to promote this trend are mainly related to reinvestment in the territory of the information collected through the devices’ sensors in the urban fabric, for instance, for sustainability purposes. From this perspective, a central-monolithic architecture would not be the optimal solution for an increasing number of devices generating data at high rates. For this purpose, the introduction of a (decentralized) mobile edge layer, at least for MCS campaigns aimed at an off-line data analytic, can mitigate possible bottlenecks and, at the same time, reduce the impact to the final user in terms of data traffic plan, battery consumption and device usage. On the other hand, studies on the interplay between mobile edges, end-devices and back-end servers are still in early phases, and the trade-offs concerning energy, latency, number of M2ECs are still to be analysed in depth. In fact, with respect to the examples of edge-based MCS paradigms discussed in the literature so far, both MCS and MEC are still in their infancy and further investigations are required before an effective, efficient, satisfactory, and possibly standardized urban sensing architecture can be reached, able to process almost real-time mobility sensing data without burden on the users’ devices’ performance and/or negatively affecting the computational support provided by the middleware MEC proxies. Concerning the needs of MCS applications, we highlighted how the social context of the end-users and their communities play an important role, and, from this perspective, they might be enhanced with social amplification strategies. Recent and more advanced MCS applications are able to autonomously select and trigger the most pertinent action, as well as the appropriate device that is best suited for a given task only on the basis of the inner circle of a subject. The higher the request of fine-grained data, the higher the need of accurate strategies for the recruitment of volunteers provided with the potential to retrieve such data. In Industry 5.0 scenarios, as well as in urban areas, the analysis of the benefits of MCS are still ongoing projects. The MCS applied to the industrial life-cycle has the potential to positively affect the time-to-market and the business plans of the industrial apparatus, as well as to ease the discovery process of the laws leading the insiders’ interactions within their workspaces. Based on these premises, we expect that in the near future, the MCS paradigm will act as a powerful tool to understand complex phenomena in industrial scenarios and urban areas with an accuracy never seen before. There may be plenty of data in the users’ pockets, but there is no room for missed data.

## References

[1] Capponi A., Fiandrino C., Kantarci B., Foschini L., Kliazovich D., Bouvry P. (2019). A Survey on Mobile Crowdsensing Systems: Challenges, Solutions, and Opportunities. IEEE Commun. Surv. Tutor..

[2] Ganti R.K., Ye F., Lei H. (2011). Mobile crowdsensing: Current state and future challenges. IEEE Commun. Mag..

[3] Alvear O., Calafate C.T., Cano J.-C., Manzoni P. (2018). Crowdsensing in smart cities: Overview platforms and environment sensing issues. Sensors.

[4] Schaffers H., Komninos N., Pallot M., Trousse B., Nilsson M., Oliveira A. (2011). Smart cities and the future Internet: Towards cooperation frameworks for open innovation. The Future Internet assembly (LNCS 6656).

[5] Zanella A., Bui N., Castellani A., Vangelista L., Zorzi M. (2014). Internet of Things for smart cities. IEEE Internet Things J..

[6] Kiukkonen N., Blom J., Dousse O., Gatica-Perez D., Laurila J. Towards Rich Mobile Phone Datasets: Lausanne Data Collection Campaign. Proceedings of the ACM International Conference on Pervasive Services (ICPS 2010).

[7] Laurila J.K., Gatica-Perez D., Aad I., Blom J., Bornet O., Do T.-M.-T., Dousse O., Eberle J., Miettinen M. The Mobile Data Challenge: Big Data for Mobile Computing Research. Proceedings of the on the Mobile Data Challenge Workshop (MDC) in Conjunction with Pervasive.

[8] Falaki H., Lymberopoulos D., Mahajan R., Kandula S., Estrin D. A first look at traffic on smartphones. Proceedings of the 10th ACM SIGCOMM Conference on Internet Measurement (IMC).

[9] Jaimes L.G., Vergara-Laurens I., Raij A. (2015). A survey of incentive techniques for mobile crowd sensing. IEEE Internet Things J..

[10] Ota K., Dong M., Gui J., Liu A. (2018). QUOIN: Incentive mechanisms for crowd sensing networks. IEEE Netw..

[11] Christin D., Reinhardt A., Kanhere S.S., Hollick M. (2011). A survey on privacy in mobile participatory sensing applications. J. Syst. Softw..

[12] Gong W., Zhang B., Li C. (2018). Task Assignment in Mobile Crowdsensing: Present and Future Directions. IEEE Netw..

[13] Eagle N., Pentland A.S. (2006). Reality mining: Sensing complex social systems. J. Pers. Ubiquitous Comput..

[14] Aslak U., Rosvall M., Lehmann S. (2018). Constrained information flows in temporal networks reveal intermittent communities. Phys. Rev. E.

[15] O’Keeffe K.P., Anjomshoaa A., Strogatz S.H., Santi P., Ratti C. (2019). Quantifying the sensing power of vehicle fleets. Proc. Natl. Acad. Sci. USA.

[16] Wang R.Q., Mao H., Wang Y., Rae C., Shaw W. (2018). Hyper-resolution monitoring of urban flooding with social media and crowdsourcing data. Comput. Geosci..

[17] Malleson N., Vanky A., Hashemian B., Santi P., Verma S.K., Courtney T.K., Ratti C. (2018). The characteristics of asymmetric pedestrian behavior: A preliminary study using passive smartphone location data. Trans. GIS.

[18] Alessandretti L., Aslak U., Lehmann S. (2020). The scales of human mobility. Nature.

[19] Pappalardo L., Simini F., Rinzivillo S., Pedreschi D., Giannotti F., Barabási A.L. (2015). Returners and explorers dichotomy in human mobility. Nat. Commun..

[20] Barbosa H., Barthelemy M., Ghoshal G., James M.L.C.R., Louail T., Menezes R., Ramasco J.J., Simini F., Tomasini M. (2018). Human mobility: Models and applications. Phys. Rep..

[21] Barbosa H., de Lima-Neto F.B., Evsukoff A., Menezes R. (2015). The effect of recency to human mobility. EPJ Data Sci..

[22] Feng J., Li Y., Zhang C., Sun F., Meng F., Guo A., Jin D. Deepmove: Predicting human mobility with attentional recurrent networks. Proceedings of the 2018 World Wide Web Conference.

[23] Blondel V.D., Decuyper A., Krings G. (2015). A survey of results on mobile phone datasets analysis. EPJ Data Sci..

[24] Lane N.D., Miluzzo E., Lu H., Peebles D., Choudhury T., Campbell A.T. (2010). A survey of mobile phone sensing. IEEE Commun. Mag..

[25] Khan W.Z., Xiang Y.M., Aalsalem A.Y.Q. (2013). Mobile phone sensing systems: A survey. IEEE Commun. Surv. Tutor..

[26] Moreira-Matias L., Gama J., Ferreira M., Mendes-Moreira J., Damas L. (2013). Predicting taxi–Passenger demand using streaming data. IEEE Trans. Intell. Transp. Syst..

[27] Pappalardo L., Ferres L., Sacasa M., Cattuto C., Bravo L. (2020). An individual-level ground truth dataset for home location detection. arXiv.

[28] Zheng Y. (2015). Trajectory Data Mining: An Overview. ACM Trans. Intell. Syst. Technol..

[29] Zheng Y., Zheng Y., Zhou X. (2011). Location-Based Social Networks: Users. Computing with Spatial Trajectories.

[30] Jurdak R., Zhao K., Liu J., AbouJaoude M., Cameron M., Newth D. (2015). Understanding Human Mobility from Twitter. PLoS ONE.

[31] Potortì F., Crivello A., Girolami M., Traficante E., Barsocchi P. Wi-Fi probes as digital crumbs for crowd localization. Proceedings of the 7th International Conference on Indoor Positioning and Indoor Navigation (IPIN).

[32] Zheng Y., Zhang L., Xie X., Ma W.-Y. Mining interesting locations and travel sequences from GPS trajectories. Proceedings of the 18th international World Wide Web Conference (WWW 2009).

[33] Zheng Y., Li Q., Chen Y., Xie X., Ma W.-Y. (2008). Understanding Mobility Based on GPS Data. Proceedings of the 10th International Conference on Ubiquitous Computing.

[34] Chessa S., Girolami M., Foschini L., Ianniello R., Corradi A., Bellavista P. (2016). Mobile crowd sensing management with the ParticipAct living lab. Pervasive Mob. Comput..

[35] Stopczynski A., Sekara V., Sapiezynski P., Cuttone A., Madsen M.M., Larsen J.E., Lehmann S. (2014). Measuring large-scale social networks with high resolution. PLoS ONE.

[36] Pappalardo L., Simini F., Barlacchi G., Pellungrini R. (2019). Scikit-mobility: A python library for the analysis, generation and risk assessment of mobility data. arXiv.

[37] Song C., Qu Z., Blumm N., Barabási A.L. (2010). Limits of Predictability in Human Mobility. Science.

[38] Génois M., Barrat A. (2018). Can co-location be used as a proxy for face-to-face contacts?. EPJ Data Sci..

[39] Guha S., Rastogi R., Shim K. (2000). Rock: A robust clustering algorithm for categorical attributes. Inf. Syst..

[40] Mcpherson M., Smith-lovin L., Cook J.M. (2001). Birds of a Feather: Homophily in Social Networks. Annu. Rev. Sociol..

[41] Rossetti G., Cazabet R. (2018). Community discovery in dynamic networks: A survey. ACM Comput. Surv. (CSUR).

[42] Javed M.A., Younis M.S., Latif S., Qadir J., Baig A. (2018). Community detection in networks: A multidisciplinary review. J. Netw. Comput. Appl..

[43] Mohamed E.M., Agouti T., Tikniouine A., El Adnani M. (2019). A comprehensive literature review on community detection: Approaches and applications. Procedia Comput. Sci..

[44] Belli D., Chessa S., Foschini L., Girolami M. (2020). The rhythm of the crowd: Properties of evolutionary community detection algorithms for mobile edge selection. Pervasive Mob. Comput..

[45] Ester M., Kriegel H.-P., Sander J., Xu X. (1996). A density-based algorithm for discovering clusters in large spatial databases with noise. Proceedings of the Second International Conference on Knowledge Discovery and Data Mining (KDD-96).

[46] David A., Vassilvitskii S. (2007). k-means++: The advantages of careful seeding. Proceedings of the Eighteenth Annual ACM-SIAM Symposium on Discrete Algorithms.

[47] Aslak U., Alessandretti L. (2020). Infostop: Scalable stop-location detection in multi-user mobility data. arXiv.

[48] Rossetti G., Pappalardo L., Pedreschi D., Giannotti F. (2017). Tiles: An onlne algorithm for community discovery in dynamic social networks. Mach. Learn..

[49] Cazabet R., Amblard F., Hanachi C. Detection of overlapping communities in dynamical social networks. Proceedings of the IEEE Second International Conference on Social Computing.

[50] Edler D., Bohlin L. (2017). Mapping higher-order network flows in memory and multilayer networks with infomap. Algorithms.

[51] Fortunato S. (2010). Community detection in graphs. Phys. Rep..

[52] Ryan M. (2020). In defence of digital contact-tracing: Human rights, South Korea and Covid-19. Int. J. Pervasive Comput. Commun..

[53] Ferretti L., Wymant C., Kendall M., Zhao L., Nurtay A., Abeler-Dörner L., Parker M., Bonsall D., Fraser C. (2020). Quantifying SARS-CoV-2 transmission suggests epidemic control with digital contact tracing. Science.

[54] Jo W., Chang D., You M., Ghim G.H. (2021). A social network analysis of the spread of COVID-19 in South Korea and policy implications. Sci. Rep..

[55] Cuttone A., Lehmann S., González M.C. (2018). Understanding predictability and exploration in human mobility. EPJ Data Sci..

[56] Cintia P., Pappalardo L., Rinzivillo S., Fadda D., Boschi T., Giannotti F., Chiaromonte F., Bonato P., Fabbri F., Penone F. (2020). The relationship between human mobility and viral transmissibility during the COVID-19 epidemics in Italy. arXiv.

[57] Prabhu S.M., Subramanyam N., Girdhar R. (2021). Containing COVID-19 Pandemic using Community Detection. J. Phys. Conf. Ser..

[58] Gibbs H., Nightingale E., Liu Y., Cheshire J., Danon L., Smeeth L., Pearson C.A.B., Grundy C., Kucharski A.J., Eggo R.M. (2021). Detecting behavioural changes in human movement to inform the spatial scale of interventions against COVID-19. PLoS Comput. Biol..

[59] Yang G., He S., Shi Z., Chen J. (2017). Promoting cooperation by the social incentive mechanism in mobile crowdsensing. IEEE Commun. Mag..

[60] Girolami M., Chessa S., Foschini L., Ianniello R., Corradi A. Social Amplification Factor for Mobile Crowd Sensing: The ParticipAct Experience. Proceedings of the IEEE International Symposium on Computers and Communications.

[61] Taleb T., Samdanis K., Mada B., Flinck H., Dutta S., Sabella D. (2017). On multi-access edge computing: A survey of the emerging 5G network edge cloud architecture and orchestration. IEEE Commun. Surv. Tutor..

[62] Belli D., Chessa S., Foschini L., Girolami M. (2020). A Probabilistic Model for the Deployment of Human-enabled Edge Computing in Massive Sensing Scenarios. IEEE Internet Things J..

[63] Aslam F., Aimin W., Li M., Rehman K.U. (2020). Innovation in the era of IoT and industry 5.0: Absolute innovation management (AIM) framework. Information.

[64] Svertoka E., Saafi S., Rusu-Casandra A., Burget R., Marghescu I., Hosek J., Ometov A. (2021). Wearables for industrial work safety: A survey. Sensors.

[65] Guo Y., Li Y., Sun Y. Accurate indoor localization based on crowd sensing. Proceedings of the 12th International Wireless Communications and Mobile Computing Conference (IWCMC).

[66] Coluccia A., Fascista A. (2019). A Review of Advanced Localization Techniques for Crowdsensing Wireless Sensor Networks. Sensors.

[67] Luca M., Barlacchi G., Lepri B., Pappalardo L. (2020). A Survey on Deep Learning for Human Mobility. arXiv.

[68] Ahmed I., Ahmad M., Ahmad A., Jeon G. (2021). IoT-based crowd monitoring system: Using SSD with transfer learning. Comput. Electr. Eng..

[69] Patrikakis C.Z., Kogias D.G., Chatzigeorgiou C., Kalyvas D., Katsadouros E., Giannousis C. A method for measuring urban space density of people and deliver notification, with respect to privacy. Digest of Technical Papers, Proceedings of the IEEE International Conference on Consumer Electronics, Virtual Conference, 10–12 January 2021.

